# Discovery of Novel Antitumor Small-Molecule Agent with Dual Action of CDK2/p-RB and MDM2/p53

**DOI:** 10.3390/molecules29030725

**Published:** 2024-02-04

**Authors:** Zhaofeng Liu, Yifei Yang, Xiaohui Sun, Runchen Ma, Wenjing Zhang, Wenyan Wang, Gangqiang Yang, Hongbo Wang, Jianzhao Zhang, Yunjie Wang, Jingwei Tian

**Affiliations:** 1 School of Pharmacy, Key Laboratory of Molecular Pharmacology and Drug Evaluation, Ministry of Education, Collaborative Innovation Center of Advanced Drug Delivery System and Biotech Drugs in Universities of Shandong, Yantai University, Yantai 264005, China; m17852656495@163.com (Z.L.); yangyifei@luye.com (Y.Y.); sxh163yx2022@163.com (X.S.); celtics5920@163.com (R.M.); wangwenyan@luye.com (W.W.); oceanygq@ytu.edu.cn (G.Y.); hongbowang@ytu.edu.cn (H.W.); zhangjianzhao@163.com (J.Z.); 2 R & D Center, Luye Pharma Group Ltd., Yantai 264003, China; zhangwenjing@luye.com

**Keywords:** antitumor drug, CDK2, MDM2, p53

## Abstract

Cell cycle-dependent kinase 2 (CDK2) is located downstream of CDK4/6 in the cell cycle and regulates cell entry into S-phase by binding to Cyclin E and hyper-phosphorylating Rb. Proto-oncogene murine double minute 2 (MDM2) is a key negative regulator of p53, which is highly expressed in tumors and plays an important role in tumorigenesis and progression. In this study, we identified a dual inhibitor of CDK2 and MDM2, III-13, which had good selectivity for inhibiting CDK2 activity and significantly reduced MDM2 expression. In vitro results showed that III-13 inhibited proliferation of a wide range of tumor cells, regardless of whether Cyclin E1 (CCNE1) was overexpressed or not. The results of in vivo experiments showed that III-13 significantly inhibited proliferation of tumor cells and did not affect body weight of mice. The results of the druggability evaluation showed that III-13 was characterized by low bioavailability and poor membrane permeability when orally administered, suggesting the necessity of further structural modifications. Therefore, this study provided a lead compound for antitumor drugs, especially those against CCNE1-amplified tumor proliferation.

## 1. Introduction

Cancer is one of the leading causes of death worldwide in both developing and developed economies. With the aging of the population and changes in lifestyle and dietary habits, the incidence and mortality rates of cancer are increasing year by year, and it is estimated that by 2025, there will be more than 20 million new cases [1,2]. Surgical resection is a treatment available for limited early-stage cancers, but its effectiveness in the treatment of advanced cancers is limited. The development of chemotherapy and molecularly targeted therapeutic agents has brought new hope for cancer treatment [3,4].

Cell cycle-dependent kinases (CDKs, including CDK4, CDK6, CDK2, and CDK1) are the central mechanism driving the cell cycle, and of the CDK-Cyclin complex, only CDK1 is universally required, whereas CDK2, CDK4, and CDK6 are necessary only for reproduction and genetics [5]. Tumor cells have to meet the requirements of rapid proliferation, so abnormal cell cycle activity occurs in almost all tumor types [6]. Therefore, the development of antitumor drugs targeting the CDK family has been a hot topic of research. The successful clinical use of CDK4/6 inhibitors also confirms that targeting cell cycle-associated proteins may be an effective antitumor strategy [7,8,9]. Although CDK4/6 inhibitors play an important role in the treatment of breast cancer, studies have shown that a subset (10% to 20%) of hormone receptor-positive (HR+), human epidermal growth factor 2-negative (HER2−) breast cancer patients are inherently insensitive to CDK4/6 inhibitors and that between 70% and 80% of CDK4/6 inhibitor-sensitive patients will develop refractory status after 12 to 36 months of treatment [10,11]. In these patients, Cyclin E1 (CCNE1) mRNA overexpression is associated with relative resistance to Palbociclib [11]. It has been reported that simultaneous inhibition of CDK2, CDK4, and CDK6 extended control of the tumor cell cycle [12]. CDK2 is an attractive target because its aberrant activation, through formation of non-canonical complexes or Cyclin E amplification [11,13], promotes resistance to CDK4/6 inhibitors, although its function is compensated by CDK1 in CDK2 knockout mice [14,15]. Therefore, CDK2 inhibitors may have a better therapeutic role in this subset of patients who are resistant to CDK4/6 inhibitors.

P53 is an important tumor suppressor that is involved in DNA repair, apoptosis, senescence, and cell cycle [16,17,18,19]. In more than half of human cancers, p53 is mutated or decreased [20,21,22], resulting in loss of tumor suppressor function or enhancement of tumor growth [23,24]. It has been shown that p53 proteins undergo up to 50 post-translational modifications, and are usually inactivated by ubiquitination by a number of E3 ubiquitin ligases [25]. The proto-oncogene murine double minute 2 (MDM2) is the major E3 ubiquitin ligase involved in proteasomal degradation [26]. It is an intracellular molecule with multiple biological functions that limits p53-mediated cell cycle block and apoptosis and promotes tissue inflammation [27]. In addition, p53 also activates MDM2, thus forming a negative feedback loop [16]. Therefore, the development of antitumor drugs targeting MDM2/p53 is a feasible approach from the perspective of activating p53 and inducing cancer cell death.

PF-07104091 (hereinafter referred to as PF-4091) is a CDK2 inhibitor developed by Pfizer, Inc. and currently undergoing clinical studies, with preclinical and clinical studies demonstrating its efficacy and safety [5,28]. In order to obtain more effective antitumor drugs, the present study explored the binding of PF-4091 to CDK2 protein molecules, and a new compound **III-13** was obtained by replacing the left methyl fragment by introducing differently substituted phenyl hydrophobic groups. Our results showed that the inhibitory activity of III-13 on CDK2 was similar to that of PF-4091, whereas the inhibitory effect on CDK1 was much weaker, and there is virtually no inhibitory activity on CDK4 and CDK6. The above results suggested that III-13 might be an effective and safer compound. More excitingly, both in vivo and in vitro results showed that III-13 had a more potent antitumor proliferation effect with a favorable safety profile. And mechanistic studies suggested that, in addition to its inhibitory effect on CDK2, III-13 significantly inhibited MDM2 mRNA and protein expression, which in turn promoted p53 protein expression, and might explain the excellent antitumor effects of III-13. In summary, this study identified an antitumor compound acting on CDK2 and MDM2/p53, preliminarily analyzed its antitumor activity and pharmacokinetic characteristics, and provided new lead compounds and ideas for the development of antitumor drugs.

## 2. Results

### 2.1. Results

#### 2.1.1. III-13 Exhibits Comparable CDK2 Inhibitory Activity and Weaker CDK1 Inhibition Effect to PF-4091

**Figure 1:**
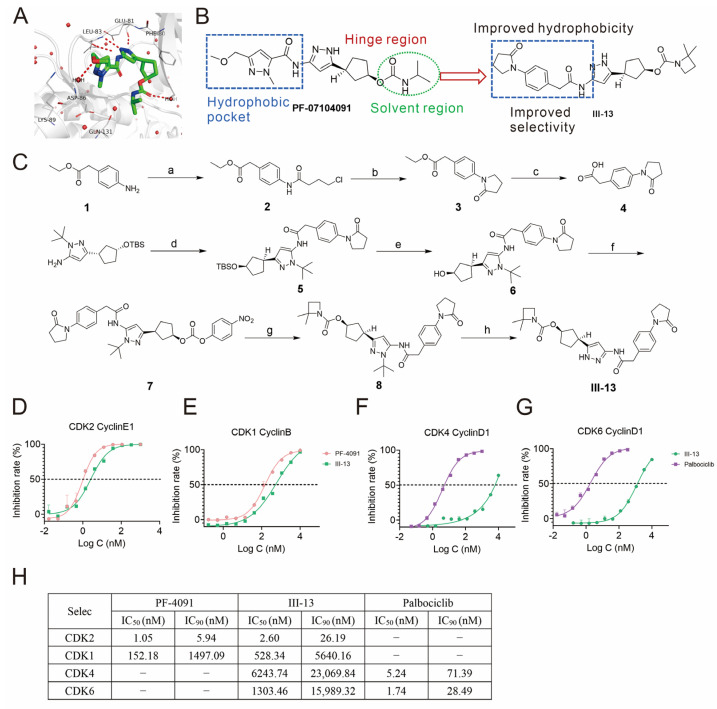
Structure modification, synthesis, and CDK inhibitory activity of III-13. (**A**) Molecular docking of PF-4091 and CDK2. (**B**) Design structure of III-13. (**C**) III-13 synthesis step. (**D**–**H**) Summary of the inhibitory effect of III-13 on CDKs. Conditions and Reagents. Letters: (a) 4-Chlorobutyryl chloride, Et3N, DCM, 0−20 °C, 16 h; (b) NaH, THF, 0−25 °C, 2 h; (c) NaOH, THF/EtOH, 60 °C, 1 h; (d) 4, TCFH, NMI, DMF, 20−70 °C, 3 h; (e) 1M HCl, MeOH, THF (10.0 V), 20 °C, 16 h; (f) 4-nitrophenyl carbonochloridate, Py, DMAP, THF, 75 °C, 22 h; (g) 2,2-dimethylazetidine, DIEA, 50 °C, 14 h; (h) TFA, DCM, 80 °C, 14 h.

As shown in Figure 1A, modeling of molecular docking of PF-4091 with CDK2 protein showed that the methyl pyrazole moiety on the left side of PF-4091 occupies the hydrophobic pocket formed by VAL18, ASP145, ALA144, and ILE10 in CDK2 protein. As shown in Figure 1B, we introduced differently substituted phenyl hydrophobic groups to replace the left methyl fragment to enhance the binding ability of the molecule to the protein as well as to improve the CDK1/CDK2 selectivity, and obtained compound **III-13**. To analyze the inhibitory effect of III-13 on CDKs, we determined the inhibitory activity of III-13 on CDK1, CDK2, CDK4, and CDK6. The half maximal inhibitory concentration (IC_50_) of III-13 inhibiting CDK2 activity was 2.60 nM, which was similar to the inhibitory activity of PF-4091 (1.05 nM) (Figure 1D,H). Excitingly, III-13 weakly inhibited CDK1 with an IC_50_ of 528.34 nM, and the IC_50_ of PF-4091 for CDK1 was 152.18 nM (Figure 1E,H). The inhibitory effects of III-13 on CDK4 and CDK6 were also determined, and the results suggested that III-13 exerted little inhibitory effect on CDK4 and CDK6 activity (Figure 1F–H). The above results suggested that III-13 was comparable in inhibiting tumor proliferation and may have a better safety profile compared to PF-4091.

#### 2.1.2. III-13 Has a Robust Inhibitory Effect on Tumor Cell Proliferation, Regardless of CCNE1 Expression

**Figure 2:**
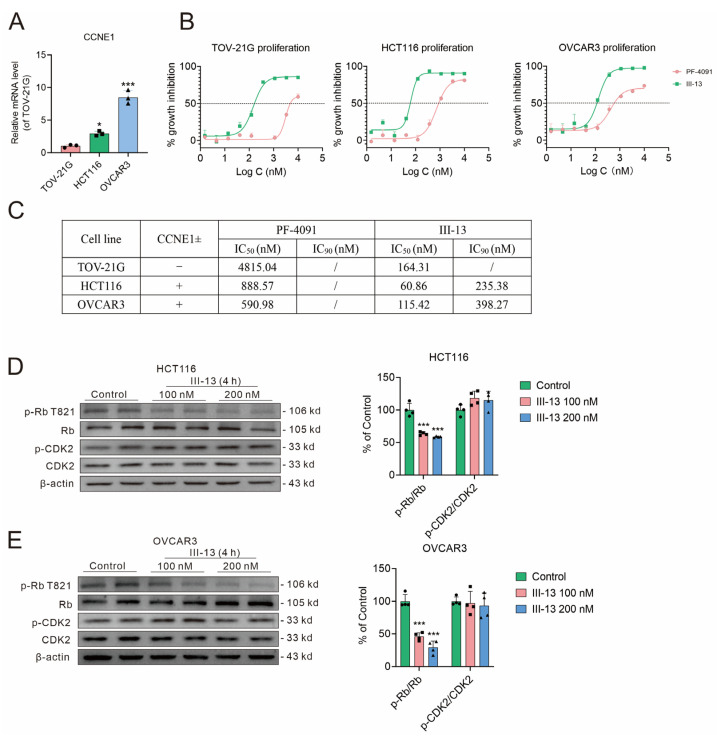
Antitumor effects of III-13 and its effect on CDK2 pathway. (**A**) *CCNE1* mRNA expression in TOV-21G, HCT116, and OVCAR3 cells. Results are expressed as mean ± SD, n = 3. * *p* < 0.05 vs. TOV-21G cells. (**B**,**C**) Inhibition of TOV-21G, HCT116, and OVCAR3 cell proliferation by III-13. (**D**,**E**) Effect of III-13 on p-Rb, Rb, p-CDK2, and CDK2 protein expression in HCT116 and OVCAR3 cells. Results are expressed as mean ± SD, n = 4. *** *p* < 0.001 vs. control group.

A previous study has shown that CDK2 inhibitor was more sensitive to inhibit CCNE1-expanded tumor cells [29]. In order to compare the antitumor activity of III-13 and PF-4091, TOV-21G (normal expression of CCNE1), HCT116 (overexpression of CCNE1), and OVCAR3 (overexpression of CCNE1) were used (Figure 2A). As shown in Figure 2B,C, the antitumor cell proliferation ability of III-13 was superior to that of PF-4091 regardless of CCNE1 expression.

Then, the effect of III-13 on CDK2 pathway was also examined. III-13 significantly reduced the expression of p-Rb, downstream of CDK2, in HCT116 and OVCAR3 cells, but had no significant effect on p-CDK2 expression (Figure 2D,E). The above results suggested that the antitumor effect of III-13 might be exerted by inhibiting CDK2 activity and its downstream p-Rb expression.

#### 2.1.3. III-13 Also Promotes P53 Activation by Inhibiting MDM2

**Figure 3:**
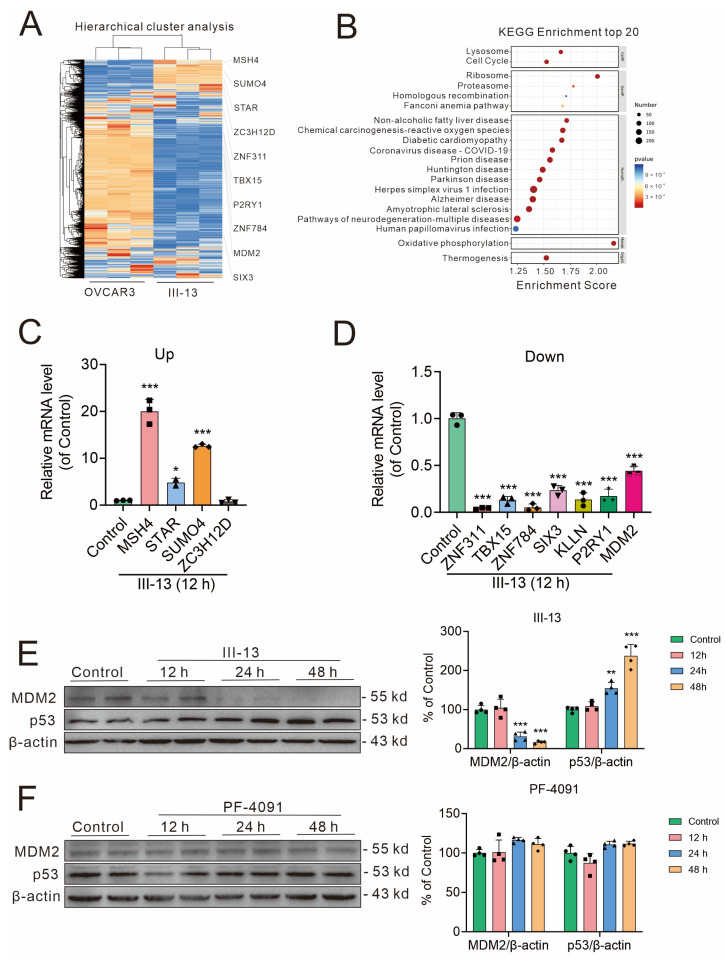
Transcriptome sequencing and related validation. (**A**) RNA sequencing cluster analysis chart. OVCAR3 cells were treated with III-13 or DMSO for 12 h. Orange color indicates gene up-regulation (*p* < 0.05 and fold change > 2) and blue color indicates gene down-regulation (*p* < 0.05 and fold change < 0.5). (**B**) KEGG signaling pathway that was significantly changed by III-13 treatment. Different colors of bubbles represent different *p* values, and different sizes of bubbles represent the number of genes in the signaling pathway. (**C**,**D**) q-PCR was performed to detect changes in mRNA after 12 h of III-13 treatment. (**E**) Changes in MDM2 and p53 protein levels after III-13-treated cells were detected by Western blot. (**F**) PF-4091 did not affect MDM2 and p53 expression in OVCAR3 cells. Results are expressed as mean ± SD, n = 3 or 4. * *p* < 0.05, ** *p* < 0.01, *** *p* < 0.001 vs. control group.

In vitro antitumor tests showed that III-13 significantly inhibited the proliferation of CCNE1-expressing normal and expanded tumor cells, which suggested that III-13 had other possible regulatory pathways. To further analyze the potential mechanism of the antitumor effects of III-13, we analyzed the transcriptome of III-13-treated OVCAR3 cells. The RNA sequencing cluster analysis plot showed that 3573 genes were up-regulated and 3893 genes were down-regulated after III-13 treatment (Figure 3A). Subsequently, we performed KEGG enrichment analysis to screen for significantly related KEGG pathways (Figure 3B). The differential genes were closely associated with cell cycle; impacts on other pathways were also evident. Next, the top 20 up-regulated or down-regulated mRNAs, which could be translated into protein, were further validated (Figure 3C,D). Among the above mRNAs, MDM2 has the most significant association with tumor survival. Therefore, we next analyzed whether III-13 exerts its antitumor effects through the MDM2 pathway. Unsurprisingly, III-13 not only significantly inhibited MDM2 mRNA expression, but also inhibited MDM2 protein expression (Figure 3D,E). As a downstream target of MDM2, p53 expression was also significantly elevated by III-13 (Figure 3E). In contrast, PF-4091 basically did not affect the expression of MDM2 and p53 (Figure 3F). Based on the above results, the antitumor effects of III-13 may be achieved through the CDK2/p-Rb and MDM2/p53 pathways, whereas PF-4091 does not affect MDM2/p53.

#### 2.1.4. III-13 Blocks Cell Cycle Progression, Inhibits Tumor Cell Migration and Colony Formation

**Figure 4:**
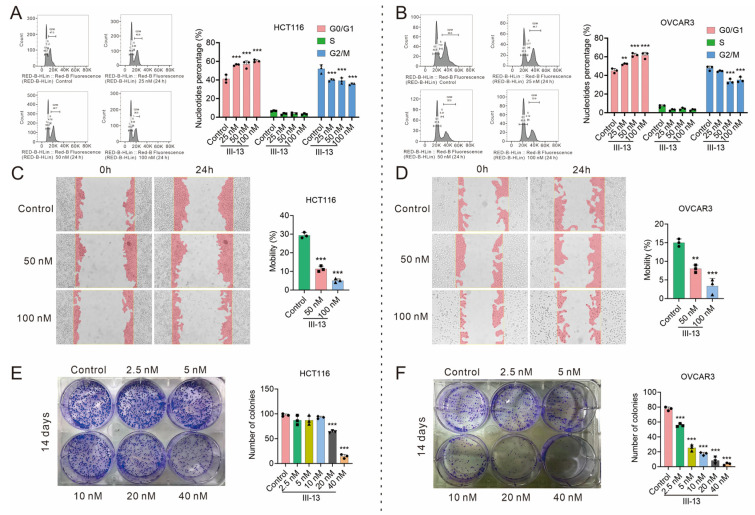
III-13 blocked cycle progression and inhibited cell migration and colony formation in HCT116 and OVCAR3 cells. (**A**,**B**) Effect of III-13 on the cell cycle of HCT116 and OVCAR3 cells detected by flow cytometry. (**C**,**D**) The effect of III-13 on cell migration was detected by scratch test. (**E**,**F**) Detection of the effect of III-13 on colony formation. Results are expressed as mean ± SD, n = 3. ** *p* < 0.01, *** *p* < 0.001 vs. control group.

CDK2 is a key kinase in the G1 phase of the cell cycle, and inhibition of CDK2 leads to cell cycle arrest, whereas MDM2 and p53 have effects on apoptosis, cell cycle arrest, cellular senescence, and autophagy in tumor cells [16]. Therefore, we used flow cytometry to further validate the cell cycle blocking site, cell colony formation assay to investigate the biological processes of tumor cell proliferation, differentiation, and apoptosis, and scratch assay to assess the invasion and migration ability of tumor cells, in order to enrich the indexes for the assessment of antitumor activity of III-13 in vitro. The results showed that III-13 arrested HCT116 and OVCAR3 cells in the G0/G1 phase (Figure 4A,B), which might be related to the inhibitory effect of III-13 on CDK2. The migration rates of HCT116 and OVCAR3 cells decreased significantly after III-13 stimulation (Figure 4C,D). As shown in Figure 4E,F, III-13 caused a significant reduction in the number of colonies of HCT116 and OVCAR3 cells. In conclusion, III-13 has good antitumor effects in vitro, and can significantly reduce the proliferation, invasion, and migration of cells.

#### 2.1.5. III-13 Demonstrates In Vivo Antitumor Activity

**Figure 5:**
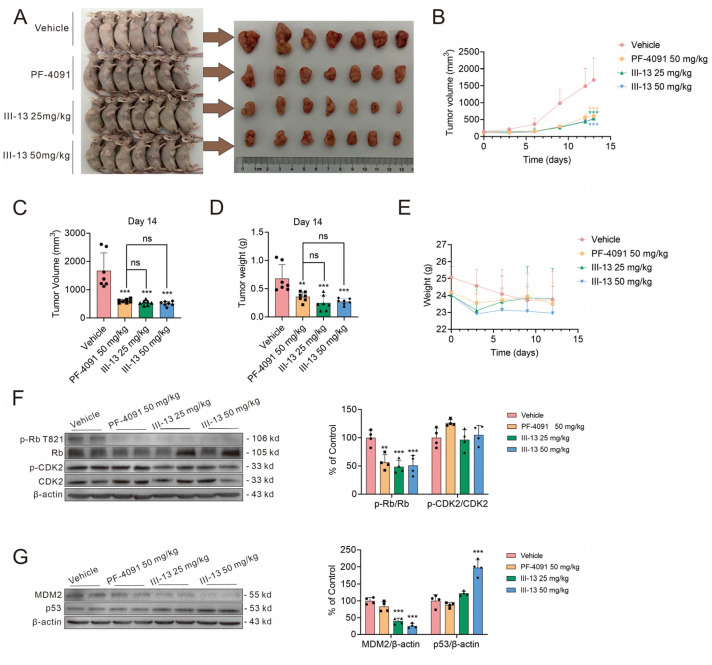
Antitumor activity of III-13 in vivo. (**A**) Comparison of nude mice and tumor volumes between groups after 14 days of compound treatment. (**B**–**E**) Quantitative plot of tumor volume, tumor weight, and body weight change. Results are expressed as mean ± SD, n = 7. (**F**,**G**) Changes in p-Rb, Rb, p-CDK2, CDK2, MDM2, and p53 protein levels in transplanted tumors in each group. Results are expressed as mean ± SD, n = 4. ** *p* < 0.01, *** *p* < 0.001 vs. vehicle group.

To further investigate the in vivo antitumor activity of III-13, we established a nude mouse model of HCT116 transplanted tumor. After 14 days of treatment, the nude mice were executed and the tumors were excised for subsequent analysis. Tumor volume in the III-13 group was markedly reduced, and when concentration reached 50 mg/kg, the tumor volume was reduced by nearly 69.81% and tumor weight was reduced by nearly 60.16%, which was similar to the effect of PF-4091 (Figure 5A–D). In addition, to analyze the safety of III-13, body weights of nude mice were also recorded. As shown in Figure 5E, body weight loss of nude mice was less than 10% in all groups. Overall, in vivo antitumor assay showed that III-13 significantly inhibited the proliferation of tumor with a good safety profile.

Subsequently, we extracted protein samples from transplanted tumor tissues of nude mice for Western blot, and the results are shown in Figure 5F,G. When nude mice with transplanted tumors were treated with PF-4091 and III-13, the level of p-Rb downstream of CDK2 in the transplanted tumors was significantly reduced, which demonstrated that both PF-4091 and III-13 exerted antitumor effects by acting on CDK2 and downstream p-Rb (Figure 5F). Moreover, the level of MDM2 was significantly down-regulated and the level of p53 protein downstream of MDM2 was up-regulated in transplanted tumors treated with III-13; meanwhile, PF-4091 had no effect on MDM2 and p53 (Figure 5G). These results further demonstrated that III-13 exerted antitumor effects through the CDK2/p-Rb and MDM2/p53 pathways.

#### 2.1.6. Evaluation of the Druggability of III-13

To further analyze the druggability of III-13, we determined the pharmacokinetic profile, membrane permeability, hepatic microsomal metabolism, and cardiotoxicity of III-13. After a single intravenous administration of III-13 (2 mg/kg), the mean elimination half-life (T_1/2_) was ~0.37 h, Area under Curve (AUC_last_) was ~2052 h·nM. After a single intragastric administration of III-13 (5 mg/kg), the time-to-peak concentration (T_max_) was ~0.25 h, T_1/2_ was ~0.84 h, AUC_last_ was ~293 h·nM, and oral bioavailability was ~3.57% (Table 1).

The membrane permeability and microsomal metabolism of III-13 were also determined. III-13 showed a slight decrease in membrane permeability (Table 2) and an accelerated clearance rate in human, mouse, and rat liver microsomes (Table 3) compared to PF-4091. The hERG assay showed no cardiotoxicity of III-13 and PF-4091 at a maximum concentration of 30 μM (Table 4). In summary, the results showed that druggability of III-13 needs to be further optimized.

## 3. Discussion

The causes of cancer are complex, including lifestyle habits, exposure to carcinogens, and heredity. The treatment of various cancers is particularly complex due to the diversity of cancer-causative factors, and different cancer types have different mutated genes, which brings limitations to the application of antitumor drugs. With the rise of immunotherapy and combination therapies, the application of cancer therapies has expanded somewhat and patient resistance has been addressed [30,31]. However, this does not fundamentally solve the problem of drug resistance, and only through the continuous research and development of new antitumor drugs can we enrich the therapeutic means. Mutations in cell cycle-related proteins are necessary for tumor cells to meet the requirements for rapid proliferation, so the development of antitumor drugs targeting the cell cycle has been a hot research topic in recent years. With the successful development and widespread clinical application of cell cycle-related inhibitors, the issue of drug resistance has been revisited. Palbociclib, the most successful cell cycle-related inhibitor, has emerged as a non-negligible drawback after its widespread clinical application [10,32]. High levels of CCNE1 have been documented to be associated with low sensitivity and resistance to CDK4/6 inhibitors [11,33]. Meanwhile, CCNE1 showed overexpression in a variety of tumors and was associated with poor prognosis [34]. Thus, targeting CCNE1 may be effective not only for CDK4/6 inhibitor-resistant tumors, but also for a wide range of CCNE1-amplified tumors.

CDK2 is strongly associated with proliferation in specific cancer types [35,36], and since CDK2 binds to cell cycle protein E to promote cell cycle progression, targeting CDK2 may offer new options for the treatment of these human cancers. The aim of this study was to develop a novel CDK2 inhibitor to provide a new therapeutic option for cancer patients with CCNE1 overexpression and resistance to CDK4/6 inhibitors. First, we structurally modified PF-4091 to obtain compound **III-13**, with a view to obtaining better antitumor activities (Figure 1A). In this study, the inhibitory effect of III-13 on the CDK family was determined. Surprisingly, III-13 showed remarkable selectivity for CDK2. Specifically, III-13 inhibited CDK2 with an IC_50_ of 2.60 nM, and its IC_50_ for CDK1, CDK4, and CDK6 reached 528.34 nM, 6243.74 nM, and 1303.46 nM, respectively. The results of Western blot also showed that III-13 stimulation significantly reduced p-RB protein expression. The above results suggested that III-13 had prominent selectivity for CDK2 and thus might not trigger unexpected adverse reactions due to selectivity for other CDKs.

Since CDK2 inhibitors had been shown to have better sensitivity in CCNE1-amplified tumors, we selected CCNE1-amplified and normal-expressing cells for the validation of III-13 and PF-4091 efficacy in antitumor proliferation assay. PF-4091, a single CDK2 inhibitor, significantly inhibited the proliferation of CCNE1-expanding cells (HCT116 and OVCAR3), but weakly inhibited the proliferation of TOV-21G cells with normal CCNE1 expression. This result is consistent with the idea that inhibition of CDK2 activity is an effective treatment for CCNE1-amplified tumors. However, although the inhibitory effect of III-13 on CDK2 was similar to that of PF-4091 and was more selective for CDK1, III-13 exhibited higher inhibitory activity on HCT116, OVCAR3, and TOV-21G cells. This result suggested that III-13 might also play a role in other antitumor targets. In view of the consideration of the safety of multi-target drugs [37,38,39], we carried out the validation of in vivo antitumor activity of III-13 and initially evaluated the safety of III-13 by the changes in body weight of mice. As shown in Figure 5, both 25 and 50 mg/kg III-13 significantly reduced tumor volume, and the effect of 25 mg/kg III-13 in reducing tumor proliferation was similar to that of 50 mg/kg PF-4091. The above results were similar to those of in vitro antitumor assay, in which III-13 inhibited the proliferation of tumor cells more effectively than PF-4091. More surprisingly, III-13 treatment did not cause significant weight loss in mice, suggesting that III-13 had a favorable safety profile although it might exert its antitumor effects through multiple targets. However, it should not be ignored that although CDK2 inhibitors can treat patients with CDK4/6 resistance, CDK2 inhibitors will still have resistance problems during the treatment process because CDK1 can partially complement CDK2 function for Rb hyper-phosphorylation, which requires that CDK2 inhibitors be used in combination with CDK4/6 inhibitors or other antitumor drugs to maximize improvement in the problem of resistance to antitumor drugs in the process of clinical use of drugs [5].

**Figure 6:**
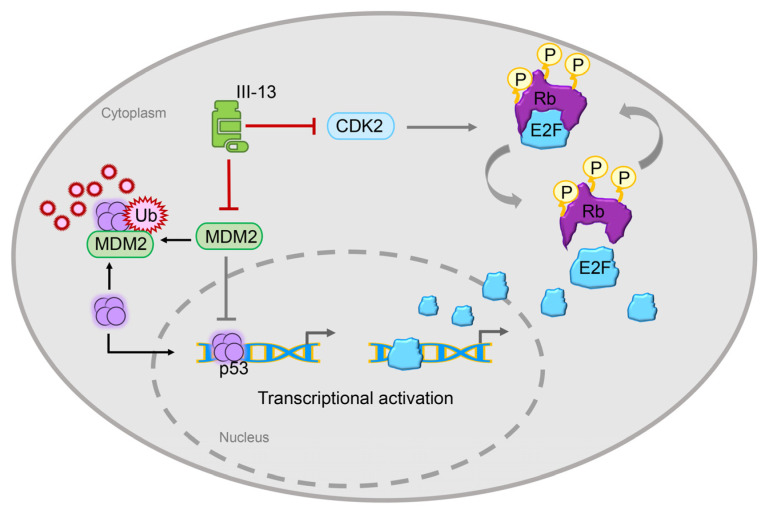
Schematic diagram. III-13 exerts antitumor effects by inhibiting the hyper-phosphorylation of Rb by CDK2, arresting tumor cells in the G0/G1 phase and significantly inhibiting the expression of MDM2, thereby increasing the expression of p53.

To search for possible targets of III-13 other than CDK2, the transcriptome of III-13-treated OVCAR3 cells was employed. Of all the mRNAs raised or lowered after III-13 treatment, change in MDM2 was the most pronounced and was associated with antitumor proliferative effects. Therefore, we determined the mRNA levels and protein levels of MDM2 after III-13 treatment. As expected, III-13 treatment resulted in a significant decrease in mRNA and protein levels of MDM2. MDM2 is a transcription factor that is oncogenic when highly expressed and spontaneously leads to tumorigenesis [40], and it has been reported to be overexpressed in many human tumors and is associated with infiltration, metastasis, and poor prognosis of many malignant tumors [41]. More importantly, MDM2 is a direct target of p53 transcriptional activation, which is a key oncogene in human cells. MDM2 binds directly to the acid-activated and partially transcription-activated structural domains of the p53 protein to form the p53-MDM2 complex, thereby inhibiting p53-mediated transcriptional activation [42,43]. At the same time, MDM2 is also a bridge between two tumor suppressors, p53 and Rb [44,45,46], which also suggested that inhibitors acting on CDK affect MDM2, which in turn exerts some antitumor effects through p53. In the present study, p53 expression was also determined after III-13 treatment. The results showed that p53 expression was significantly reduced at 48 h and 72 h after III-13 stimulation. The above results fully indicated that III-13 exerted antitumor effects through the CDK2/p-Rb pathway and MDM2/p53 pathway (Figure 6).

Cell cycle progression assay, cell migration assay, and colony formation assay were used to analyze the multiple antitumor effects of III-13. Results showed that III-13 arrested HCT116 and OVCAR3 cells in G0/G1 phase, and significantly inhibited the migration and colony formation of HCT116 and OVCAR3 cells. The above results suggested that III-13 exerted its antitumor effects by inducing cell cycle arrest, and inhibiting cell migration and clone formation. Finally, we performed a preliminary evaluation of the drug-forming properties of III-13. We found that III-13 had a shorter T_1/2_, worse bioavailability, and poorer membrane permeability after oral administration compared to PF-4091. This implied that the pharmacokinetic behavior of III-13 needs to be further improved in order to have a certain degree of druggability.

## 4. Materials and Methods

### 4.1. Reagent

The following antibodies were used in the study: phospho-Rb T821 (Thermo Fisher Scientific, Waltham, MA, USA, 1:1000 dilution), Rb (Abcam, Cambridge, UK, 1:2000 dilution), phosphor-CDK2 T160 (Cell Signaling Technology, Danvers, MA, USA, 1:1000 dilution), CDK2 (Abcam, Cambridge, UK, 1:1000 dilution), Cyclin E1 (Proteintech, Wuhan, China, 1:1000 dilution), p53 (Proteintech, Wuhan, China, 1:5000 dilution), MDM2 (Santa Cruz Biotechnology, Dallas, TX, USA, 1:1000 dilution), and β-actin (Beyotime, Shanghai, China, 1:1000 dilution).

PF-07104091 and Compound **III-13** were synthesized by WuXi AppTec (Shanghai, China).

### 4.2. Animals

Adult male Balb/c nude mice (6–8 weeks old, initial weight 18–22 g) were purchased from Beijing Huafukang Biotechnology Co., Ltd. (Beijing, China). Adult male and female rats (4–5 weeks old, initial weight 180–200 g) were purchased from Beijing Viton Lihua Laboratory Animal Technology Co., Ltd. (Beijing, China). The mice and rats were housed in groups with a 12/12 h light/dark cycle and were allowed to drink and eat freely. The study was approved by the Laboratory Animal Ethics Committee of Yantai University, China, and complied with the 2011 Guide for the Care and Use of Laboratory Animals (Institute for Laboratory Animal Resources in the Life Sciences, National Research Council of the National Academy of Sciences, Washington, DC, USA).

### 4.3. Cell Culture

The HCT116 cell line was purchased from ATCC (Manassas, VA, USA), and cells were cultured in RPMI-1640 (Gibco, Grande Island, NY, USA) media supplemented with 10% fetal bovine serum (Gibco, USA) and 1% penicillin–streptomycin (Beyotime, Shanghai, China). The OVCAR3 cell line was purchased from Procell (Wuhan, China); the cells were cultured in RPMI-1640 media supplemented with 20% fetal bovine serum and 1% penicillin–streptomycin. The TOV-21G cell line was purchased from Cobioer Biosciences (Nanjing, China); the cells were cultured in TOV-21G complete medium (Cobioer Biosciences, Nanjing, China) and incubated at 37 °C in a humidified atmosphere containing 5% CO_2_. All cells were harvested during the exponential growth phase for assays.

### 4.4. Molecular Docking

To better understand the binding mode of PF-4091 to the CDK2 receptor, we performed molecular docking studies using the Glide program (version 10.2, Schrodinger) as previously described. These protein structures were preprocessed using the Protein Preparation Wizard of Schrödinger Maestro to prepare for molecular docking using default settings. Water molecules were retained in the crystals and missing residues in the crystal structures were automatically reconstructed. Finally, the structures were subjected to constrained energy minimization using the Optimized Potentials for Liquid Simulations (OPLS) 2005 force field. The active site of the target was defined by the original ligand. The ligands were docked using the Glide program, using the generated grid file with active site residues. The structural sketches of the ligands were saved in mol2 format. The compounds were subjected to ligand preparation by the Ligprep wizard application and ionized states were generated at pH 7.0 ± 2.0. All structures were minimized using the OPLS3 force field. The van der Waals radius was set to 0.8 with a partial cutoff of 0.15. Docking was performed using the Glide module in the Schrodinger suite. The best docking attitude was chosen as the one with the lowest Glide score. 

### 4.5. Synthesis of Compound **III-13**

Compound **1** (5.00 g, 1.00 *eq*), triethylamine (5.56 g, 2.00 *eq*), was dissolved sequentially in 50 mL CH_2_Cl_2_. The mixture was suspended at 0 °C, and then compound **1A** (4.33 g, 1.10 *eq*) was added. Then, the reaction mixture was stirred at 20 °C for 16 h. TLC monitored the reaction until the starting material was completely consumed. H_2_O (100 mL) was added and extracted with CH_2_Cl_2_ (3 × 400 mL). The combined CH_2_Cl_2_ layer was dried over sodium sulfate and concentrated in vacuo. The crude residue was purified by silica gel column chromatography to give compound **2** as a yellow solid (7.80 g) with 75.5% purity.

Compound **2** (7.00 g, 1.00 *eq*) was dissolved in THF (70 mL), and then NaH (1.18 g, 60.0% purity, 1.20 *eq*) was slowly added to the reaction in batches. Then, the reaction mixture was stirred at 25 °C for 2 h. TLC monitored the reaction until the starting material was completely consumed. Saturated NH_4_Cl solution (100 mL), H_2_O (60 mL) was added and extracted with ethyl acetate (2 × 100 mL). The combined ethyl acetate layer was dried over sodium sulfate and concentrated in vacuo. The crude residue was purified by silica gel column chromatography to give compound **3** as a yellow solid (3.80 g) with 62.2% yield, 95.3% purity.

Anhydrous ethanol (20 mL), compound **3** (2.00 g, 1.00 *eq*), NaOH (1.0 M, 20.6 mL, 2.55 *eq*), was dissolved sequentially in 40 mL THF. Then, the reaction mixture was stirred at 60 °C for 1 h. TLC monitored the reaction until the starting material was completely consumed, and then the reaction solution was concentrated in vacuo. H_2_O (20 mL) was added and extracted with ethyl acetate (50 mL). Then, 1 M HCl was added slowly with stirring until the pH of the solution reached 3~4. The precipitate obtained through filtration is the crude product compound **4A** as a yellow solid (1.40 g) with 78.9% yield, 75.5% purity.

Compound **4** (649 mg, 1.00 *eq*), 1-(tert-butyl)-3-((1S,3R)-3-((tert-butyldimethylsilyl)oxy)cyclopentyl)-1H-pyrazol-5-amine (1.00 g, 1.00 *eq*), NMI (729 mg, 3.00 *eq*), was dissolved sequentially in DMF (10 mL). Then, the reaction mixture was stirred at 70 °C for 3 h. TLC monitored the reaction until the starting material was completely consumed. H_2_O (150 mL) was added and extracted with ethyl acetate (3 × 70 mL). The combined ethyl acetate layer was dried over sodium sulfate and concentrated in vacuo. The crude residue was purified by silica gel column chromatography to give compound **5** as a red solid (600 mg) with 30.1% yield, 80.0% purity.

Compound **5** (600 mg, 1.00 *eq*), HCl (1.0 M, 6.00 mL, 5.39 *eq*), was dissolved sequentially in methanol (6 mL). Then, the reaction mixture was stirred at 20 °C for 16 h. TLC monitored the reaction until the starting material was completely consumed. Then, the reaction solution was concentrated in vacuo to give compound **6** as a red solid (570 mg).

Compound **6** (570 mg, 1.00 *eq*, HCl salt), compound **6A** (373 mg, 1.50 *eq*), pyridine (293 mg, 3.00 *eq*), DMAP (15.1 mg, 0.10 *eq*), was dissolved sequentially in THF (7 mL). Then, the reaction mixture was stirred at 75 °C for 22 h. TLC monitored the reaction until the starting material was completely consumed. H_2_O (6 mL) was added and extracted with ethyl acetate (3 × 6 mL). The combined ethyl acetate layer was dried over sodium sulfate and concentrated in vacuo. The crude residue was purified by thin-layer chromatography to give compound **7** as a yellow oil (320 mg) with 43.8% yield, 13.5% purity.

Compound **7** (320 mg, 1.00 *eq*, HCl), compound **7A** (99.0 mg, 2.50 *eq*, HCl salt), DIEA (210 mg, 3.00 *eq*), was dissolved sequentially in THF (3 mL). Then, the reaction mixture was stirred at 50 °C for 14 h. TLC monitored the reaction until the starting material was completely consumed. H_2_O (5 mL) was added and extracted with ethyl acetate (3 × 4 mL). The combined ethyl acetate layer was dried over sodium sulfate and concentrated in vacuo. The crude residue was purified by silica gel column chromatography to give compound **8** as a colorless oil (90 mg) with 30.9% yield.

Compound **8** (30 mg, 1.00 *eq*), TFA (0.30 mL, 26.40 *eq*) was dissolved sequentially in THF (3 mL). Then, the reaction mixture was stirred at 80 °C for 16 h. TLC monitored the reaction until the starting material was completely consumed, and then the reaction solution was concentrated in vacuo. The crude residue was purified by silica gel column chromatography to give compound **III-13** as a white solid (11.0 mg) with 36.4% yield, 92.0% purity. ^1^H NMR (400 MHz, Chloroform-*d*) *δ*: 8.37 (s, 1H), 7.56 (d, *J* = 8.0 Hz, 2H), 7.26 (d, *J* = 8.0 Hz, 2H), 6.47 (s, 1H), 5.10 (s, 1H), 3.81 (t, *J* = 6.8 Hz, 2H), 3.63 (s, 2H), 3.40 (t, *J* = 6.8 Hz, 2H), 3.20 (t, *J* = 6.8 Hz, 1H), 2.60 (t, *J* = 8.0 Hz, 2H), 2.46–2.39 (m, 1H), 2.19–1.93 (m, 4H), 1.91–1.76 (m, 3H), 1.64 (t, *J* = 6.0 Hz, 2H), 1.32 (s, 6H) (Supplementary Materials).

### 4.6. Kinase Antagonist Activity Assay

The compounds were solubilized with DMSO (Solarbio Life Sciences, Beijing, China), and 11 concentrations were serially diluted at 3-fold; 100 nL of compound was added to 384-well plates, 5 μL of 2 × enzyme mixture (Thermo Fisher Scientific, Waltham, MA, USA) was added, and 5 μL of buffer was added instead of the complete inhibition control wells. Then, 1000 rpm centrifugation was performed for 30 s followed by a 15 min incubation at 23 °C. Next, 5 μL of 2 × MLight-MBP peptide (PerkinElmer, Waltham, MA, USA) was added to each well, with 1000 rpm centrifugation for 30 s and incubation at 23 °C for 90 min. At the end of the incubation, the reaction was terminated with buffer containing 15 mM EDTA and 2 nM Eu-anti-P-MBP antibody (PerkinElmer, Waltham, MA, USA), centrifuged at 1000 rpm for 1 min, and incubated at 23 °C for 60 min; the plates were read on a Perkin Elmer Envision (Bio Tek, Winooski, VT, USA). TR-FRET ratio is calculated by Envision automatically (the value of 665 nm/the value of 620 nm), and TR-FRET ratio is normalized to calculate % inhibition: (100% inhibition − sample data)/(100% inhibition − 0% inhibition) × 100.

### 4.7. Cell Proliferation Assay

Celltiter-Glo Luminescent Cell Viability Assay (Promega, Madison, WI, USA) was used to detect cell viability according to the manufacturer’s instructions. This assay is a homogeneous method of determining the number of viable cells in a culture based on quantitation of the ATP present, an indicator of metabolically active cells. Cells were digested and paved in white-bottomed 96-well plates with 1000 cells/100 μL cell suspension solution; then, different concentrations of compounds were added the next day. TOV-21G and HCT116 were placed in the incubator for three days and OVCAR3 for five days. Then, 100 μL Celltiter-Glo solution was added and mixed for 5 min; the luminous intensity of each well was detected with a spectrophotometer (BioTek, Winooski, VT, USA). The experiment was repeated three times to calculate the cell survival rate.

### 4.8. Western Blot

Total protein from HCT116, OVCAR3 cells and tissues was collected and homogenized in RIPA buffer. The total cellular protein extract was electrophoresed on 12% SDS-polyacrylamide gels and then transferred to a PVDF membrane (Thermo Fisher Scientific, Waltham, MA, USA). Primary antibodies were incubated overnight at 4 °C and secondary antibodies were incubated at room temperature for 1.5 h. Imaging was performed using Image Quant LAS4000 (GE, Boston, MA, USA).

### 4.9. Cell Cycle Detection

Cells were plated at 300,000 cells overnight in six-well plates. III-13 was added in a dose-dependent fashion and then cells were incubated for 24 h. The cells were then fixed with 75% ethanol overnight at 4 °C. The cells were centrifuged, and ethanol was removed. The cells were washed twice with 1 × PBS following staining with DAPI (4′, 6′ diamino-2-phenylindole) and analyzed using Guava easyCyte HT (Merck KGaA, Darmstadt, Germany) with a blue filter. Experiments were performed in triplicate.

### 4.10. Cell Scratch Test

Cells were inoculated into six-well plates as long as they spread to the bottom of the wells after wall attachment. After 24 h of wall attachment, cells were scratched with a 200 μL pipette tip and then replaced with low serum medium (1% FBS) containing different concentrations of compounds. Spot photographs were taken at 0 h and 24 h after the scratch was made. Data analysis was performed using Adobe Photoshop 2021 (Adobe Systems Software Ireland Ltd., San Jose, CA, USA) and Image J2 (National Institutes of Health, Bethesda, MD, USA).

### 4.11. Experiments in Colony Formation

Cells were inoculated in six-well plates at 1000 cells per well, and III-13 was added in a dose-dependent manner after 24 h. Cells were stained with Crystal Violet Staining Solution (Beyotime, Shanghai, China) at 14 days after incubation, and the cell colonies were counted using Image J.

### 4.12. Tumor Xenograft Experiments

HCT116 cells (1 × 10^6^) were implanted into the back of adult male Balb/c nude mice by subcutaneous injection. When the tumor volume reached 100 cubic millimeters, the mice were randomly divided into four groups (7 mice per group): vehicle group, PF-4091 group (50 mg/kg), III-13 group (25 mg/kg), and III-13 group (50 mg/kg). The solvents for PF-4091 and III-13 were 1% DMSO, 10% Solutol, and 89% saline. The solvent or drug containing solution was administered daily to the vehicle and dosing groups by intraperitoneal injection (0.2 mL/20 g). Tumor growth was measured every three days during treatment. At the end of the treatment, the mice were killed, and the tumors were removed and weighed.

### 4.13. mRNA Sequencing

OVCAR3 cells cultured overnight were treated with DMSO and III-13, and samples were collected after 12 h for reference transcriptome sequencing (Oebiotech, Shanghai, China). Total RNA was extracted using Trizol (Vazyme, Nanjing, China) and sent for sequencing. Sequence comparison of CleanReads to a specified reference genome was performed using hisat [47], and positional information was obtained on a reference genome or gene, as well as sequence characterization information specific to the sequenced sample. DESeq2 1.22.2 [48] software was used to standardize the number of counts of each sample gene (BaseMean value was used to estimate the expression amount), calculate the multiplicity of differences, test the significance of differences using NB (Negative Binomial Distribution Test), and then screen the differential protein coding genes according to the multiplicity of differences and the significance of differences test results. Pathway analysis was performed on the differential protein-coding genes using the KEGG [49] database (in conjunction with the KEGG annotation results), and the significance of differential gene enrichment in each pathway entry was calculated using a hypergeometric distribution test.

### 4.14. Real-Time Quantitative Polymerase Chain Reaction

RNA was reverse transcribed with the HiScript Q RT SuperMix for qPCR (Vazyme, Nanjing, China), and quantitative real-time PCR (qPCR) was applied using SYBR Green qPCR Mix (SparkJade, Jinan, China) on the ABI Prism 7500 Fast Real-Time PCR System (Applied Biosystems, Carlsbad, CA, USA) following the manufacturer’s protocols. The comparative CT method (ΔΔCt) was used to calculate gene expression differences, with GAPDH as the reference gene. The following primers were used (human):

*ZNF311* (sense, 5′-3′): GATGGAAGCCAAGGAAACCTGC
*ZNF311* (antisense, 5′-3′): TGCCTTTGAGCGTAGGTCAGAC
*TBX15* (sense, 5′-3′): TGACCTCTGGAAGCGGTTC
*TBX15* (sense, 5′-3′): GATGTGGATCTAGGCCAGTGA
*ZNF784* (sense, 5′-3′): GCCAGGTTCTTTCCACTGTG
*ZNF784* (antisense, 5′-3′): CCCCGTGTGCAAGCTGTAG
*SIX3* (sense, 5′-3′): CTGCCCACCCTCAACTTCTC
*SIX3* (antisense, 5′-3′): GCAGGATCGACTCGTGTTTGT
*KLLN* (sense, 5′-3′): GTTGAGTGGAAAGTACGGAACG
*KLLN* (antisense, 5′-3′): TGTGGGTGCTTGTGTAACCAG
*P2RY1* (sense, 5′-3′): GGGATGCCATGTGTAAACTGC
*P2RY1* (antisense, 5′-3′): CGCTGATACAGATCGCATTCTT
*MSH4* (sense, 5′-3′): CTGGACACCACAAGTGGGATA
*MSH4* (antisense, 5′-3′): CAGCTACAATAACTGATGGGGAG
*STAR* (sense, 5′-3′): GGGAGTGGAACCCCAATGTC
*STAR* (antisense, 5′-3′): CCAGCTCGTGAGTAATGAATGT
*SUMO4* (sense, 5′-3′): CCACGGGGATTGTCAGTGAA
*SUMO4* (antisense, 5′-3′): CCTCCCGTAGGCTGTTGAAA
*ZC3H12D* (sense, 5′-3′): AGAAACCTTCTCTTGCCGGG
*ZC3H12D* (antisense, 5′-3′): CGTGCTGCTCTCTGATAGGG
*CCNE1* (sense, 5′-3′): AAGGAGCGGGACACCATGA
*CCNE1* (antisense, 5′-3′): ACGGTCACGTTTGCCTTCC
*MDM2* (sense, 5′-3′): GAATCATCGGACTCAGGTACATC
*MDM2* (antisense, 5′-3′): TCTGTCTCACTAATTGCTCTCCT
*GAPDH* (sense, 5′-3′): GGAGCGAGATCCCTCCAAAAT
*GAPDH* (antisense, 5′-3′): GGCTGTTGTCATACTTCTCATGG

### 4.15. Evaluation of Permeability and Transporter Substrates

Caco-2 cells were cultured in MEM with 20% FBS, penicillin (100 U/mL), and streptomycin (100 μg/mL) at 37 °C in 95% air and 5% CO_2_. Cells were seeded into 96-well cell plates and cultured continuously for 24 days for subsequent experiments. III-13 (2 μM) and PF-4091 (2 μM) were added. After 120 min of incubation, samples were collected from the apical and basal ends. The urea content of each sample was determined by liquid chromatography–tandem mass spectrometry (LC-MS/MS). The apparent osmotic coefficient and efflux ratio were calculated.

### 4.16. Evaluation of Liver Microsomal Stability In Vitro

Compounds were incubated with mouse, rat, and human liver microsomal samples and NADPH at 37 °C for 5, 10, 20, 30, or 60 min. The reaction was terminated with a cold acetonitrile solution containing tolbutamide. Compound retention was determined by LC-MS/MS. The retention times, chromatographic acquisition, and chromatographic integration of compounds and positive control substances were analyzed using Analyst (AB Sciex, Framingham, MA, USA).

### 4.17. Human Ether-à-Go-Go-Related Gene (hERG) Assay

This test method is a reference to a previous study [50]. CHO cells stably expressing hERG were cultured with medium containing 10% FBS, penicillin (100 U/mL), and streptomycin (100 μg/mL) in DMEM/F12 in an incubator at 37 °C, 5% CO_2_. Cultures were passaged in a 1:5 ratio every 48 h. The medium consisted of 90% F12, 10% FBS, G418 (100 μg/mL), and hygromycin B (100 μg/mL). On the day of the assay, the current should reach a steady state for at least 1 min prior to administration of the compounds, and the test current was measured again after 5 min of stimulation with different concentrations of the drug.

### 4.18. Pharmacokinetics Study

The pharmacokinetics study was created as described [51]. PF-4091 and III-13 were administered via intragastric (5 mg/kg) and intravenous (2 mg/kg) routes to female and male rats. Approximately 200 μL of whole blood was collected into heparin-coated tubes at 0.083, 0.25, 0.5, 1, 2, 3, 4, 6, 8, 12, and 24 h after dosing. Plasma was collected immediately by centrifugation (8000× *g*, 5 min, room temperature). Blood was placed in a heparinized Eppendorf™ tube. Plasma was centrifuged, and the concentration of compounds in the sample was determined by LCMS/MS after pre-treatment. Drug-concentration data were processed in a non-atrioventricular model using WinNonlin™ 6.3 (Pharsight, Mountain View, CA, USA).

### 4.19. Statistical Analysis

All statistical analyses were performed using GraphPad Prism 9 Software (La Jolla, CA, USA). Data were expressed as the mean ± SD. Statistical differences among groups were analyzed by using one-way ANOVA followed by Tukey’s post hoc test. * *p* < 0.05; ** *p* < 0.01; *** *p* < 0.001.

## 5. Conclusions

In this study, we identified a novel and potent anticancer compound, **III-13**. In vitro assays showed that it inhibited the proliferation of CCNE-expanded and non-expanded tumor cells, and the results of in vivo experiments suggested that III-13 had an excellent antitumor proliferation effect and good safety profile. Mechanistic studies revealed that III-13 was exclusively selective for CDK2 and had an effect on the MDM2/p53 pathway.

## Figures and Tables

**Table 1:** Pharmacokinetics of III-13 in rats.

Compound	T_max_ (h)	C_max_ (nM)	AUC_last_ (h·nM)	T_1/2_ (h)	V_z_/F (L/kg)	C_L_/F (L/h/kg)	MRT_last_ (h)	F (%)
III-13 (2 mg/kg. i.v.)	-	5502 ± 2307	2052 ± 337	0.37 ± 0.13	1304 ± 374	2470 ± 442	0.45 ± 0.25	-
III-13 (5 mg/kg. i.g.)	0.25	389 ± 87.9	293 ± 64.6	0.84 ± 0.12	-	-	0.85 ± 0.10	3.57
PF-4091 (2 mg/kg. i.v.)	-	6711 ± 1480	5340 ± 621	2.60 ± 0.27	3515 ± 222	944 ± 106	1.20 ± 0.18	-
PF-4091 (5 mg/kg. i.g.)	0.33 ± 0.14	3458 ± 2204	9976 ± 3409	6.37 ± 5.35	-	-	4.07 ± 1.73	46.7

**Table 2:** Membrane permeability of III-13 and PF-4091.

Compound	Mean P_app_ (10^−6^ cm/s)		Efflux Ratio	Rank	
	A to B	B to A		Papp	Efflux Transporter Substrate
III-13	1.60	8.56	5.34	47.7	52.5
PF-4091	4.3	37.0	8.57	80.5	99.3

**Table 3:** Microsomal metabolic stability in liver microsomes from different species.

Compound	T_1/2_ (min)			CL_int_ (mic) (mL/min/kg)			CL_int_ (liver) (mL/min/kg)			Remaining (%, T = 60 min)		
	Human	Mice	Rat	Human	Mice	Rat	Human	Mice	Rat	Human	Mice	Rat
III-13	21.9	6.53	28.1	63.4	212	49.4	59.9	840	88.9	15.4	1.29	22.8
PF-4091	>186	87.7	>186	<7.5	15.8	<7.5	<13.5	62.6	<6.75	97.0	61.5	90.5

**Table 4:** hERG experiments with III-13 and PF-4091.

Compound	IC_50_
III-13	30 μM
PF-4091	>30 μM

## Data Availability

Some or all data generated or analyzed during this study are included in this published article.

## Supplementary Materials

The following supporting information can be downloaded at: https://www.mdpi.com/article/10.3390/molecules29030725/s1, Figure S1: ^1^H NMR (400 MHz, Chloroform-*d*) spectrum of compound **III-13**: *δ* 8.37 (s, 1H), 7.56 (d, *J* = 8.0 Hz, 2H), 7.26 (d, *J* = 8.0 Hz, 2H), 6.47 (s, 1H), 5.10 (s, 1H), 3.81 (t, *J* = 6.8 Hz, 2H), 3.63 (s, 2H), 3.40 (t, *J* = 6.8 Hz, 2H), 3.20 (t, *J* = 6.8 Hz, 1H), 2.60 (t, *J* = 8.0 Hz, 2H), 2.46–2.39 (m, 1H), 2.19–1.93 (m, 4H), 1.91–1.76 (m, 3H), 1.64 (t, *J* = 6.0 Hz, 2H), 1.32 (s, 6H).
